# Impact of sex, age, and ethnicity/race on the survival of patients with rectal cancer in the United States from 1988 to 2012

**DOI:** 10.18632/oncotarget.10696

**Published:** 2016-07-19

**Authors:** Martin D. Berger, Dongyun Yang, Yu Sunakawa, Wu Zhang, Yan Ning, Satoshi Matsusaka, Satoshi Okazaki, Yuji Miyamoto, Mitsukuni Suenaga, Marta Schirripa, Annika Medea Lenz, Pierre Bohanes, Afsaneh Barzi, Jane C. Figueiredo, Diana L. Hanna, Heinz-Josef Lenz

**Affiliations:** ^1^ Division of Medical Oncology, Norris Comprehensive Cancer Center, Keck School of Medicine, University of Southern California, Los Angeles CA, USA; ^2^ Department of Preventive Medicine, Norris Comprehensive Cancer Center, Keck School of Medicine, University of Southern California, Los Angeles, CA, USA

**Keywords:** age, ethnicity/race, sex, rectal cancer, survival

## Abstract

Most studies report on colon and rectal cancers collectively, even though biologic and prognostic differences exist between these disease entities. Here, we investigated the effects of sex, age, and ethnicity/race on rectal cancer (RC) mortality by stage focusing on differences before and after 2004.

Using the SEER database, we identified 105,511 patients diagnosed with RC from 1988-2012. Main outcomes were disease-specific survival (DSS) and overall survival (OS).

In patients with stage I-III RC, women achieved a longer DSS (HR 0.87, *P* < 0.001) than men, independent of age, from 1988-2012. In stage IV disease, the sex disparity favoring women was limited to the age 18-44 yr cohort (DSS HR 0.79, *P* < 0.001). The sex difference in DSS (*P*_interaction_ = 0.009) was significantly reduced from 2004 to 2012 across all ages. Hispanics and Native Americans with locoregional RC had inferior DSS relative to Whites from 1988-2003, but these differences were not evident from 2004-2012 (*P*_interaction_ = 0.001). Additionally, Asians with stage I-III RC had superior DSS from 2004 on compared to Whites. Mortality in African American patients improved modestly overall and remained significantly higher than other ethnicities/races across all stages.

Sex disparities have narrowed in patients with metastatic RC, but persist in patients with stage I-III disease. These differences are most evident among young patients (18-44 years), where sex disparities have even widened in stage I-III disease. While outcomes have improved for Asians, Hispanics, and Native Americans with stage I-III rectal cancer, black-white disparities remain in all disease stages.

## INTRODUCTION

Rectal cancer (RC) is a leading cause of morbidity and mortality from gastrointestinal malignancy [1]. While cancer-specific survival has improved in the United States [2], demographic disparities remain for reasons which are incompletely understood. For instance, there has been a steady rise in the incidence and mortality of RC among individuals younger than 50 years old [3–5]. Indeed, younger patients are more likely to present with rectal than proximal colon tumors, and with poorly differentiated and late-stage cancers [6].

With regards to ethnicity/race, existing evidence has shown consistently worse outcomes for African Americans compared to Whites, even after adjusting for socioeconomic and insurance status [7, 8]. However, these findings are restricted to patients with colorectal cancer (CRC), whereas outcome data specific for RC are lacking.

Over the last three decades, the management of RC has witnessed the introduction of several cytotoxic and biologic drugs, as well as the shifting of perioperative chemoradiotherapy from the adjuvant to the neoadjuvant setting for locally advanced disease [9]. In 2004, the use of oxaliplatin was expanded into the adjuvant and first-line metastatic settings, and the first targeted agents were approved for metastatic disease (i.e. bevacizumab, cetuximab) [10–13]. Whether these advancements have variably influenced outcomes in different populations has been largely un-explored.

Furthermore, although survival disparities in RC have been identified, many clinical and epidemiologic studies have examined colon and rectal cancers collectively or concentrated on a particular demographic or time period [6, 14–17]. A comprehensive investigation specific to RC and spanning recent changes in standard of care is lacking. Here, we sought to evaluate the impact and interactions of sex, age, and ethnicity/race on the survival of patients with RC. We then compared stage-specific survival trends by year of diagnosis, focusing on differences before and after 2004.

## RESULTS

### Patient, tumor, and treatment characteristics

Among 105,511 patients diagnosed with rectal cancer between 1988 and 2012 in this study, 58.5% were male and 41.5% female (Table 1). The median age at diagnosis was 66 years, and 54.3% of patients were 65 years or older. The cohort included 74.5% Whites (n = 78,593), 8% Asians (n = 8,403), 8% African Americans (n = 8,473), 8.9% Hispanics (n = 9,388), and 0.6% Native Americans (n = 654). Hispanics and Native Americans were diagnosed at a younger age than Whites. Here, the greatest difference is seen between Native American (median 60 years) and white women (median 69 years) (*P* < 0.001 for all comparisons) (Supplementary Table 1). Among Whites, African Americans and Asians, women were diagnosed at an older age than men (69 vs 66 yrs, 64 vs 62 yrs, and 65 vs 64 yrs, respectively, *P* < 0.001 for all comparisons) (Supplementary Table 1).

**Table 1:** Characteristics of patients with rectal cancer, SEER 1988-2012

	All Patients	Stage I-III	Stage IV
**Characteristic**	*N* = 105,511	*N* = 88,491	*N* = 17,020
**Sex**			
Male	61693 (58.5%)	51252 (57.9%)	10441 (61.3%)
Female	43818 (41.5%)	37239 (42.1%)	6579 (38.7%)
**Age, years**			
Median (interquartile range)	66 (56-76)	67 (56-76)	64 (54-74)
18-44	6903 (6.5%)	5438 (6.1%)	1465 (8.6%)
45-54	16786 (15.9%)	13697 (15.5%)	3089 (18.1%)
55-64	24570 (23.3%)	20329 (23.0%)	4241 (24.9%)
65-74	27720 (26.3%)	23542 (26.6%)	4178 (24.6%)
75 and older	29532 (28.0%)	25485 (28.8%)	4047 (23.8%)
**Ethnicity/Race**			
White	78593 (74.5%)	66574 (75.2%)	12019 (70.6%)
African American	8473 (8.0%)	6725 (7.6%)	1748 (10.3%)
Asian	8403 (8.0%)	7056 (8.0%)	1347 (7.9%)
Hispanic	9388 (8.9%)	7629 (8.6%)	1759 (10.3%)
Native American	654 (0.6%)	507 (0.6%)	147 (0.9%)
**Histology**			
Non-mucinous Adenocarcinoma	94735 (89.8%)	79875 (90.3%)	14860 (87.3%)
Mucinous Adenocarcinoma	6512 (6.2%)	5443 (6.1%)	1069 (6.3%)
Other	4264 (4.0%)	3173 (3.6%)	1091 (6.4%)
**Differentiation**			
Good	8456 (8.0%)	7642 (8.6%)	814 (4.8%)
Moderate	67967 (64.4%)	58791 (66.5%)	9176 (53.9%)
Poor	16509 (15.7%)	12687 (14.3%)	3822 (22.5%)
Not determined	12579 (11.9%)	9371 (10.6%)	3208 (18.8%)
**Lymph Nodes Examined**			
<12 nodes	71544 (67.8%)	58369 (66.0%)	13175 (77.4%)
≥12 nodes	31333 (29.7%)	28138 (31.8%)	3195 (18.8%)
Unknown	2634 (2.5%)	1984 (2.2%)	650 (3.8%)
**Surgery^1^**			
Not cancer-directed	18483 (17.5%)	9084 (10.3%)	9399 (55.2%)
Local	13775 (13.1%)	13251 (15.0%)	524 (3.1%)
Radical	71280 (67.5%)	64493 (72.8%)	6787 (39.9%)
Unknown	1973 (1.9%)	1663 (1.9%)	310 (1.8%)
**Radiation Sequence with Surgery^1^,^2^**			
Neoadjuvant	23020 (53.7%)	21167 (53.5%)	1853 (55.5%)
Adjuvant	18578 (43.3%)	17239 (43.6%)	1339 (40.1%)
Other sequence^3^	1291 (3.0%)	1146 (2.9%)	145 (4.4%)
**Chemotherapy^4^**			
No/unknown	50377 (47.7%)	44719 (50.5%)	5658 (33.2%)
Any	55134 (52.3%)	43772 (49.5%)	11362 (66.8%)
**Year of Diagnosis**			
1988-2003	51171 (48.5%)	43506 (49.2%)	7665 (45.0%)
2004-2012	54340 (51.5%)	44985 (50.8%)	9355 (55.0%)

1 Initial surgical procedure

2 Patients who received both initial radiation and local or radical surgery were included

3 Includes radiation both before and after surgery; intraoperative radiation +/- other radiation given before or after surgery; sequence unknown, but both surgery and radiation were performed

4 First course

The stage distribution was as follows: 34.7% stage I (n = 36,598), 23.5% stage II (n = 24,803), 25.7% stage III (n = 27,090), and 16.1% stage IV (n = 17,020). Most patients (80.6%) underwent local tumor resection or radical surgery. 53.7% received neoadjuvant and 43.3% adjuvant radiotherapy. The proportion of patients diagnosed in 1988 to 2003 and 2004 to 2012 were 48.5% (n = 51,171) and 51.5% (n = 54,340), respectively.

### Sex, age, and survival by stage and year of diagnosis

In a multivariable Cox proportional hazards regression model (Table 2), women with stage I-III RC had a significantly longer DSS (HR 0.87, 95% CI 0.84, 0.89) and OS (HR 0.78, 95% CI 0.77, 0.80) than men (*P* < 0.001). In stage IV disease, women maintained a modest advantage for OS (HR 0.96, 95% CI 0.93, 0.99, *P* = 0.019) but not for DSS (HR 0.97, 95% CI 0.94, 1.01, *P* = 0.16).

**Table 2:** Associations between patient characteristics and disease-specific and overall survival

	Stage I-III				Stage IV			
Characteristic	Disease-Specific Survival		Overall Survival		Disease-Specific Survival		Overall Survival	
	5-Year Rate	HR (95% CI)*	5-Year Rate	HR (95% CI)*	Median, months	HR (95% CI)*	Median, months	HR (95% CI)*
**Sex**								
Male	0.73	1 (reference)	0.59	1 (reference)	16	1 (reference)	14	1 (reference)
Female	0.75	0.87 (0.84, 0.89)	0.65	0.78 (0.77, 0.80)	16	0.97 (0.94, 1.01)	14	0.96 (0.93, 0.99)
*P* value*		<0.001		<0.001		0.16		0.019
**Age, years**								
18-44	0.80	0.44 (0.41, 0.47)	0.77	0.27 (0.26, 0.29)	17	0.74 (0.69, 0.80)	16	0.67 (0.63, 0.72)
45-54	0.81	0.44 (0.42, 0.46)	0.77	0.28 (0.27, 0.29)	18	0.70 (0.66, 0.74)	17	0.64 (0.60, 0.67)
55-64	0.79	0.50 (0.48, 0.52)	0.72	0.36 (0.35, 0.37)	17	0.77 (0.73, 0.81)	15	0.73 (0.69, 0.77)
65-74	0.75	0.61 (0.59, 0.63)	0.62	0.53 (0.52, 0.54)	15	0.87 (0.83, 0.92)	13	0.85 (0.81, 0.89)
75 and older	0.64	1 (reference)	0.43	1 (reference)	14	1 (reference)	11	1 (reference)
*P* value*		<0.001		<0.001		<0.001		<0.001
**Ethnicity/Race**								
White	0.75	1 (reference)	0.62	1 (reference)	16	1 (reference)	14	1 (reference)
African American	0.69	1.30 (1.24, 1.37)	0.56	1.23 (1.18, 1.27)	15	1.14 (1.08, 1.22)	13	1.15 (1.09, 1.22)
Asian	0.75	0.93 (0.88, 0.99)	0.65	0.88 (0.84, 0.92)	16	0.95 (0.88, 1.03)	14	0.98 (0.91, 1.05)
Hispanic	0.72	1.10 (1.05, 1.16)	0.61	1.02 (0.98, 1.06)	17	0.97 (0.91, 1.04)	14	0.99 (0.93, 1.05)
Native American	0.69	1.10 (0.89, 1.36)	0.55	1.18 (1.00, 1.38)	17	0.88 (0.70, 1.11)	15	0.86 (0.70, 1.07)
*P* value*		<0.001		<0.001		<0.001		<0.001

* Based on the multivariable Cox proportional hazards regression model adjusting for the variables included in the table and histology, differentiation, T stage, N stage, AJCC TNM7 stage (for stage I-III), surgery, radiation therapy, sequence of radiation to surgery, chemotherapy, number of lymph nodes resected, CEA level, year of diagnosis, marital status at diagnosis, and stratified by SEER registration sites.

With regards to age, patients younger than age 55 achieved the longest DSS (HR 0.66, 95% CI 0.64, 0.69, *P* < 0.001) and OS (HR 0.47, 95% CI 0.46, 0.49, *P* < 0.001) compared to aged 55 or older among those with stage I-III disease. In patients with stage IV rectal cancer, those aged 45-54 had the longest DSS (HR 0.70, 95% CI 0.66, 0.74, *P* < 0.001) and OS (HR 0.64, 95% CI 0.60, 0.67, *P* < 0.001) compared to patients older than 75 years (*P* < 0.001).

We then examined the interaction between age and sex on survival (Table 3). Within the stage I-III cohort, women had a significantly longer DSS (*P*_interaction_ = 0.27) and OS (*P*_interaction_ = 0.94), independent of age. In contrast, in patients with stage IV disease, the impact of sex on DSS (*P*_interaction_ = 0.013) and OS (*P*_interaction_ = 0.005) was limited to the youngest age group (18-44 years).

**Table 3:** Associations between sex and disease-specific and overall survival by age at diagnosis

Stage	Age Group	Disease-Specific Survival		Overall Survival	
		HR* (95% CI)	*P* value†	HR* (95% CI)	*P* value†
**I-III**	18-44 years	0.86 (0.77, 0.97)	0.014	0.80 (0.72, 0.89)	<0.001
	45-54 years	0.83 (0.76, 0.89)	<0.001	0.80 (0.74, 0.86)	<0.001
	55-64 years	0.83 (0.78, 0.88)	<0.001	0.78 (0.74, 0.82)	<0.001
	65-74 years	0.88 (0.83, 0.93)	<0.001	0.78 (0.74, 0.81)	<0.001
	75 and older	0.89 (0.85, 0.93)	<0.001	0.78 (0.76, 0.81)	<0.001
	*P*_for interaction_†	0.27		0.94	
**IV**	18-44 years	0.79 (0.70, 0.89)	<0.001	0.78 (0.69, 0.88)	<0.001
	45-54 years	1.01 (0.92, 1.11)	0.83	1.02 (0.94, 1.11)	0.67
	55-64 years	0.97 (0.90, 1.05)	0.45	0.96 (0.90, 1.03)	0.29
	65-74 years	0.99 (0.92, 1.07)	0.87	0.95 (0.89, 1.02)	0.15
	75 and older	1.01 (0.94, 1.08)	0.87	0.99 (0.93, 1.06)	0.86
	*P*_for interaction_†	0.013		0.005	

* Males as a reference.

† Based on Wald test in the multivariable Cox proportional hazards regression model adjusting for ethnicity/race, histology, differentiation, T stage, N stage, AJCC TNM7 stage (for stage I-III), surgery, radiation therapy, sequence of radiation to surgery, chemotherapy, number of lymph nodes resected, CEA level, year of diagnosis, marital status at diagnosis, and stratified by SEER registration sites.

**Figure 1:**
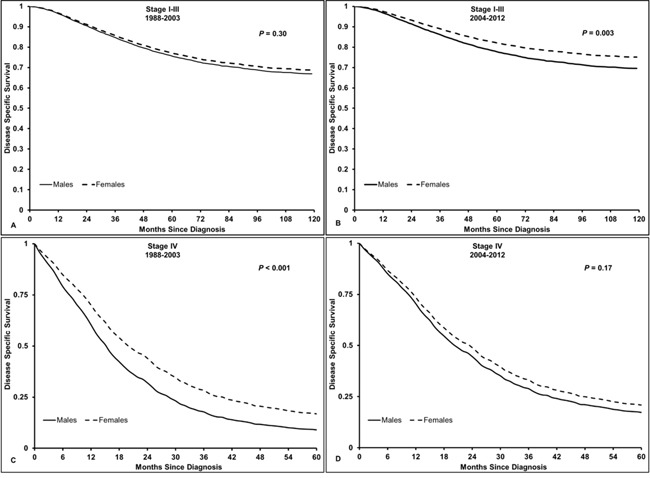
Disease-specific survival in patients with rectal cancer, aged 18-44 years old **A.** Stage I-III RC from 1988-2003, HR 0.92 (95% CI 0.79, 1.08, *P* = 0.30); **B.** Stage I-III RC from 2004-2012, HR 0.75 (95% CI 0.62, 0.90, *P* = 0.003); **C.** Stage IV RC from 1988-2003, HR 0.64 (95% CI 0.53, 0.78, *P* < 0.001); **D.** Stage IV RC from 2004-2012, HR 0.88 (95% CI 0.74, 1.06, *P* = 0.17). *P*_interaction_ stage I-III RC = 0.090, *P*_interaction_ stage IV RC = 0.018. Males used as a reference.

When stratified by year of diagnosis, the influence of sex on DSS was consistent in patients with stage I-III rectal cancer overall (*P*_interaction_ = 0.46) (Table 4). However, there was a significant interaction between sex and year of diagnosis for OS, with a narrowing of sex differences over time (*P*_interaction_ = 0.04) (Table 4). Among patients aged 18-44 years, there was a trend towards a wider sex disparity from 2004 to 2012 (DSS HR 0.75, *P*_interaction_ = 0.090; OS HR 0.73, *P*_interaction_ = 0.11) as compared to 1988 to 2003 (DSS HR 0.92, OS HR 0.88) (Figure 1, Supplementary Table 2). This trend was not evident in the other age groups. In patients with metastatic disease, the sex difference in DSS (*P*_interaction_ = 0.009) was significantly reduced from 2004 to 2012, with a similar trend for OS (*P*_interaction_ = 0.050) across all ages (Table 4).

**Table 4:** Associations between sex and disease-specific and overall survival by year of diagnosis

Stage	Year of Diagnosis	Disease-Specific Survival		Overall Survival	
		HR* (95% CI)	*P* value	HR* (95% CI)	*P* value
**I-III**	1988-2003	0.86 (0.83, 0.89)	<0.001	0.77 (0.75, 0.79)	<0.001
	2004-2012	0.88 (0.84, 0.92)	<0.001	0.81 (0.78, 0.84)	<0.001
	*P*_for interaction_†	0.46		0.040	
**IV**	1988-2003	0.93 (0.88, 0.98)	0.005	0.93 (0.88, 0.97)	0.002
	2004-2012	1.02 (0.97, 1.08)	0.40	0.99 (0.95, 1.04)	0.75
	*P*_for interaction_†	0.009		0.050	

* Males as a reference.

† Based on Wald test in the multivariable Cox proportional hazards regression model adjusting for age, ethnicity/race, histology, differentiation, T stage, N stage, AJCC TNM7 stage (for stage I-III), surgery, radiation therapy, sequence of radiation to surgery, chemotherapy, number of lymph nodes resected, CEA level, marital status at diagnosis, and stratified by SEER registration sites.

### Ethnicity/race and survival by stage and year of diagnosis

Ethnicity/race was an independent prognostic factor across all stages of rectal cancer, with the most pronounced disparities seen among African American patients (Table 2). In stage I-III disease, African American patients had a significantly shorter DSS and OS compared to Whites (DSS HR 1.30, 95% CI 1.24, 1.37; OS HR 1.23, 95% CI 1.18, 1.27, *P* < 0.001 for both comparisons), Hispanic, and Asian patients. Similarly, in stage IV disease, African Americans had an inferior DSS relative to Whites (HR 1.14, 95% CI 1.08, 1.22, *P* < 0.001), Hispanic, and Asian patients as well as a significantly shorter OS compared to all other ethnicities/races. Furthermore, Hispanics with stage I-III disease had a significantly shorter DSS (HR 1.10, 95% CI 1.05, 1.16, *P* < 0.001) compared to Whites. Asians had a modestly improved DSS and OS relative to Whites (DSS HR 0.93, 95% CI 0.88, 0.99; OS HR 0.88, 95% CI 0.84, 0.92, *P* < 0.001 for both comparisons) and Hispanics, and the longest OS amongst all ethnicities/races with stage I-III disease.

Lastly, we assessed the influence of ethnicity/race on survival across time (Table 5). African American patients had significantly shorter DSS and OS in all disease stages throughout 1988-2012. In stage I-III rectal cancer, the influence of ethnicity/race on survival varied significantly between 1988-2003 and 2004-2012 with respect to the Hispanic, Native American, and Asian cohorts. The survival differences among Hispanics and Native Americans compared to Whites in 1988-2003 were no longer evident from 2004-2012 (DSS *P*_interaction_ = 0.001, OS *P*_interaction_ = 0.058). Asian patients maintained an OS advantage relative to Whites independent of year of diagnosis, though the improvement in DSS appeared only after 2004. For patients with stage IV disease, the effect of ethnicity/race on outcomes did not differ over time.

**Table 5:** Associations between ethnicity/race and disease-specific and overall survival by year of diagnosis

	Race	Disease-Specific Survival		Overall Survival	
		HR* (95% CI)	*P* value	HR* (95% CI)	*P* value
**Stage I-III**					
1988-2003	African American	1.38 (1.29, 1.46)	<0.001	1.25 (1.19, 1.31)	<0.001
	Asian	0.96 (0.90, 1.04)	0.32	0.89 (0.84, 0.94)	<0.001
	Hispanic	1.13 (1.06, 1.21)	<0.001	1.05 (1.00, 1.11)	0.056
	Native American	1.35 (1.05, 1.74)	0.020	1.30 (1.07, 1.59)	0.010
2004-2012	African American	1.20 (1.11, 1.29)	<0.001	1.18 (1.12, 1.26)	<0.001
	Asian	0.87 (0.79, 0.95)	0.002	0.84 (0.78, 0.90)	<0.001
	Hispanic	1.05 (0.97, 1.13)	0.21	0.97 (0.91, 1.03)	0.34
	Native American	0.85 (0.63, 1.14)	0.27	1.04 (0.83, 1.29)	0.76
	*P*_for interaction_†	0.001		0.058	
**Stage IV**					
1988-2003	African American	1.18 (1.08, 1.29)	<0.001	1.18 (1.09, 1.28)	<0.001
	Asian	0.99 (0.89, 1.09)	0.79	1.01 (0.92, 1.12)	0.79
	Hispanic	1.00 (0.91, 1.10)	0.97	1.04 (0.95, 1.13)	0.40
	Native American	0.90 (0.66, 1.22)	0.49	0.86 (0.64, 1.15)	0.31
2004-2012	African American	1.10 (1.01, 1.20)	0.023	1.12 (1.04, 1.21)	0.004
	Asian	0.92 (0.83, 1.02)	0.095	0.94 (0.85, 1.03)	0.17
	Hispanic	0.94 (0.86, 1.02)	0.14	0.94 (0.87, 1.02)	0.15
	Native American	0.88 (0.65, 1.18)	0.39	0.88 (0.66, 1.16)	0.35
	*P*_for interaction_†	0.61		0.38	

* Whites as a reference.

† Based on Wald test in the multivariable Cox proportional hazards regression model adjusting for sex, age, histology, differentiation, T stage, N stage, AJCC TNM7 stage (for stage I-III), surgery, radiation therapy, sequence of radiation to surgery, chemotherapy, number of lymph nodes resected, CEA level, marital status at diagnosis, and stratified by SEER registration sites.

## DISCUSSION

Biologic, clinical, and prognostic differences distinguish rectal from colon cancer [19, 20]. Nonetheless, these two diseases are often grouped together in clinical trials and epidemiologic cohorts, and prior investigations specific to RC have been relatively limited in scope. To our knowledge, this is the largest analysis examining the impact of demographic characteristics on the survival of patients with RC. Our study revealed evolving stage-specific effects of sex, age, and ethnicity/race on RC mortality over a twenty-five year period.

Consistent with prior evidence, female sex and younger age emerged as positive prognosticators for cancer-specific and overall survival. A pooled analysis of five clinical trials in locally advanced RC have demonstrated reduced mortality in women and patients younger than 60 years of age [21]. Moreover, our findings in rectal cancer patients are in line with a previous study in colon cancer patients showing that younger women with advanced disease achieve superior outcomes, whereas post-menopausal women have a significantly shorter survival compared to age-matched men [22]. Biologically, derangements in hormonal metabolism are inherent to colorectal carcinogenesis, and sex differences might be partly attributable to the protective effect of estrogen as well as favorable immune responses seen in women after surgical resection [23–25].

Two potential contributing factors we could not account for in our study were duration of therapy and toxicity, both of which have been shown to vary by sex and predict outcomes. Retrospective analyses from CAO/ARO/AIO-94 show that although women were less likely to receive adequate radiation, they were also more likely to sustain acute toxicity from chemoradiotherapy, and such toxicity was a surrogate for improved OS [26, 27]. Similarly, results from INT-0114 demonstrate increased toxicity to adjuvant chemoradiation and improved survival in women, though toxicity was not independently associated with outcomes in this study [28].

Notably, while sex disparities significantly improved for patients with metastatic disease, they seem to be increasing in patients with early-stage RC. One possible explanation is that women not only have less intrinsically aggressive disease, but may also derive greater treatment intensity with chemoradiation or more benefit from cytotoxic chemotherapy [28]. Mechanistically, a recent translational study implicated β-tubulin as an androgen-driven mechanism of colorectal tumor aggression that is constituitively active in men, and linked to oxaliplatin and irinotecan resistance as well as poor outcomes [29]. Other pharmacogenomic data support the notion that sex modulates the sensitivity to chemotherapy [30].

The narrowing of sex outcome disparities in metastatic RC after 2004 likely reflects multiple interacting factors and the expansion of available chemotherapeutics. While we could not describe the specific chemotherapy prescribed, recent data encompassing private, academic, and community-based practice patterns from 2004 to 2011 demonstrate that men and women are equally likely to receive cetuximab or bevacizumab in the first-line metastatic setting [31].

With regards to ethnicity/race, African Americans had a significantly shorter survival in all stages of disease, which is consistent with several other studies documenting ethnic/racial disparities in cancer-associated outcomes [32–34]. African Americans have previously been reported to receive less adjuvant chemotherapy and curative treatment modalities [32, 33]. However, data from the BRiTE registry and N9741 suggest equivalent survival between African Americans and Whites, though also showed ethnic/racial differences in pharmacogenetic profiles [35, 36]. After accounting for important confounding variables, including receipt of chemotherapy, radiation, and surgery (including number of lymph nodes resected), our data demonstrated that the black-white disparities have persisted over time. These findings imply that, among other reasons, response to therapy may vary by ethnicity/race. This hypothesis is supported by previous data showing significantly lower response rates to bevacizumab-based therapy in African Americans as compared to Whites [35, 36].

In addition to African Americans, from 1988 to 2003, Hispanics and American Natives with stage I-III RC had a poorer DSS and OS compared to Whites. After 2004, the prognosis for Hispanics, Native Americans, and Asians markedly improved compared to Whites, with the greatest benefit most evident among Native Americans. This may reflect improved access to care in underserved populations.

In summary, we observe that sex disparities in outcome have narrowed in patients with metastatic RC, but persist in patients with stage I-III disease. These differences are most evident among young patients aged 18-44 years old, where sex disparities have even widened in stage I-III rectal cancer. Furthermore, younger age and female sex are associated with favorable outcome in all disease stages. In stage IV disease, the sex disparity favoring women was limited to the age 18-44 yr cohort. Hispanics and Native Americans were diagnosed at a younger age than Whites, with the greatest difference seen between Native American and white women. Among Whites, African Americans and Asians, women were diagnosed at an older age than men. These findings may have implications for future prevention strategies. While outcomes have significantly improved for Asians, Hispanics, and Native Americans with stage I-III rectal cancer, mortality in African American patients improved modestly overall and remained significantly higher than other ethnicities/races across all stages. For patients with stage IV rectal cancer, the effect of ethnicity/race on outcome did not change over time.

The main limitations of our study are the missing information regarding the type and duration of chemotherapy and radiation administered, surgical approach, presence of comorbidities and socioeconomic status, as these data are not available in the SEER registry. This renders it impossible to draw conclusions regarding the differential benefit of chemotherapeutics and biologicals in different populations. By focusing on outcome disparities before and after 2004, we were not able to systematically account for the differential effects of therapies approved at other time points. In addition, we did not have access to information on comorbidities, but by assessing DSS, we tried to limit its influence on outcomes. Nonetheless, our study represents the largest cohort of RC patients, reflects mortality data after the implementation of recent, important changes in care standards, and accounts for overall treatment rendered.

Future trials should address whether response to biologics and cytotoxic chemotherapy may vary by ethnicity/race and sex, in order to better personalize the management of patients with rectal cancer.

Additionally, the implementation of prospective population-based registries containing clinicopathologic, pharmacogenetic, and socioeconomic data will be instrumental to achieving these objectives.

## MATERIALS AND METHODS

### Study design

The SEER database 1973-2012 (Version April 2015) was used for this analysis [18]. The SEER Program, sponsored by the National Cancer Institute (NCI), provides information on the demographics, primary tumor site, tumor morphology, and stage at diagnosis, first course of treatment including surgery and radiation, and survival of all newly diagnosed cancer patients by registration site. Information on the first course of chemotherapy was obtained from NCI/SEER Program through a special request for customized data. The SEER registries expanded from 9 sites in 1973 to 18 in 2000, covering about 28% of the US population and oversampling Hispanics (38%), Native Americans (44%), Asians (50%), and Pacific Islanders (67%).

### Patient population

Patients diagnosed with rectal cancer (according to the International Classification of Diseases for Oncology, 3rd Edition [ICD-O-3] site code C20.9 and histologic type ICD-O-3 codes 8000-8152, 8154-8231, 8243-8245, 8250-8576, 8940-8950, 8980-8981) between 1988 and 2012 were identified from the SEER database (n = 135,243). Those diagnosed before 1988 (n = 33,622) were excluded due to limited staging information such as primary tumor extension, lymph node involvement, and/or distant metastasis. Other exclusion criteria included SEER historic stage A (*in situ* or un-staged disease), age (<18 or unknown), type of reporting source (autopsy only or death certificate), lack of diagnostic confirmation (not microscopically confirmed), no or unknown follow-up, and unknown ethnicity/race. The AJCC staging 7^th^ edition was used to define stage I, II, III, and IV based on primary tumor extension, lymph node involvement, and/or distant metastasis. Patients with AJCC stage 0 disease were excluded. A total of 105,511 patients met the eligibility criteria and were included in the final analysis. The patient population was further grouped into five ethnic/racial cohorts as follows: non-Hispanic Whites, non-Hispanic African Americans, non-Hispanic Asian/Pacific Islanders, Hispanics, and Native Americans. Hispanics were identified by using the North American Association of Central Cancer Registries (NAACCR) Hispanic Identification Algorithm (NHIA). Throughout the text, the term White refers to non-Hispanic Whites unless stated otherwise. Similarly, the terms African American, Asian and Native American exclude Hispanics.

Rectum (C20.9)-specific surgery codes 20 (1983-1997) and 20-28 (1998-2012) were summarized as local surgical procedures, whereas codes 30-70 (1983-1997) and 30-90 (1998-2012) were defined as radical surgery. Codes 00-10 (1983-1997) and 00, 10-14 (1998-2012) were considered as non-surgical procedures.

### Statistical analysis

The main outcomes of the study were rectal cancer or disease-specific survival (DSS) calculated from date of diagnosis to the date of death from rectal cancer and overall survival (OS), defined as the interval from date of diagnosis to the date of death. OS and DSS were censored at date of last contact, December 31, 2012, 10 years after diagnosis, or date of death from other causes for DSS, whichever came first. For subjects who were censored for OS (n = 52,587), the median follow-up time was 71 months (interquartile ranges: 30-120 months). A multivariable Cox proportional hazards regression model was used to evaluate the association between sex, age, and ethnicity/race and OS and DSS when adjusting for other potential prognostic factors available in the SEER database. We considered marital status at diagnosis, histology, tumor differentiation, tumor stage, lymph node stage, AJCC stage, the number of lymph nodes examined, surgical procedure, use of radiation therapy, sequence of surgery and radiation therapy, chemotherapy, CEA level (year of diagnosis 2004 or later), SEER registry site, and year of diagnosis as covariates in the Cox regression model. A likelihood ratio test was performed to test the interactions between sex, age, ethnicity/race, and stage on DSS and OS. We evaluated graphically departures from the proportional hazards assumption for the model using smoothed plots of weighted Schoenfeld residuals. No violation was detected. To evaluate age at diagnosis by sex and ethnicity/race, *P* values were calculated based on Wilcoxon two-sample test to compare sex differences within each ethnicity/race, whereas the Kruskal-Wallis test was used to compare ethnic/racial differences for males and females separately. Analyses for localized (AJCC stage I, II, or III) and metastatic disease (AJCC stage IV) were conducted separately using SAS Version 9.4 (SAS Institute, Cary, NC) at a significance level of 0.05. All tests were 2-sided.

## SUPPLEMENTARY MATERIALS TABLES


