# Supplementary material for: Impact of sex, age, and ethnicity/race on the survival of patients with rectal cancer in the United States from 1988 to 2012
Source: Oncotarget. 2016 Jul 19;7(33):53668–78. doi: 10.18632/oncotarget.10696 (PMC5288213; doi:10.18632/oncotarget.10696)
Supplement: Supplementary file 1 [file oncotarget-07-53668-s001.pdf]

Impact of sex, age, and ethnicity/race on the survival of patients with rectal cancer in the United States from 1988 to 2012

SUPPLEMENTARY TABLES

Supplementary Table 1: Age at diagnosis by sex and ethnicity/race

Race	Male			Female			*P* value*
	N	Median, yrs	Interquartile range	N	Median, yrs	Interquartile range	
White	45635	66	(57-75)	32958	69	(57-79)	<0.001
African American	4794	62	(53-71)	3679	64	(54-75)	<0.001
Asian	5104	64	(54-73)	3299	65	(54-75)	<0.001
Hispanic	5765	62	(52-72)	3623	62	(51-73)	0.32
Native American	395	61	(52-68)	259	60	(50-71)	0.70
P value†		<0.001			<0.001		

* Based on Wilcoxon two-sample test.
† Based on Kruskal-Wallis test.

Supplementary Table 2: Associations between sex and disease-specific and overall survival among patients 18-44 years by year of diagnosis

Stage	Year of Diagnosis	Disease-Specific Survival		Overall Survival	
		**HR* (95% CI)**	***P* value†**	**HR* (95% CI)**	***P* value†**
I-III	1988-2003	0.92 (0.79, 1.08)	0.30	0.88 (0.76, 1.01)	0.061
	2004-2012	0.75 (0.62, 0.90)	0.003	0.73 (0.61, 0.87)	<0.001
	$P_{\text{for interaction}}$†	0.090		0.11	
IV	1988-2003	0.64 (0.53, 0.78)	<0.001	0.65 (0.54, 0.78)	<0.001
	2004-2012	0.88 (0.74, 1.06)	0.17	0.86 (0.73, 1.02)	0.090
	$P_{\text{for interaction}}$†	0.018		0.025	

* Males as a reference.
† Based on Wald test in the multivariable Cox proportional hazards regression model adjusting for ethnicity/race, histology, differentiation, T stage, N stage, AJCC TNM7 stage (for stage I-III), surgery, radiation therapy, sequence of radiation to surgery, chemotherapy, number of lymph nodes resected, CEA level, marital status at diagnosis, and stratified by SEER registration sites.
